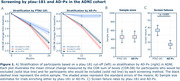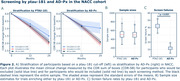# Reducing Screen Failure Rates Due to Biomarker Cut‐offs in Early AD Trials with a Prognostic Model

**DOI:** 10.1002/alz.094583

**Published:** 2025-01-09

**Authors:** Angela Tam, César Laurent, Christian Dansereau

**Affiliations:** ^1^ Perceiv AI, Montreal, QC Canada

## Abstract

**Background:**

Alzheimer’s disease (AD) trials have high screen failure rates. Recent trials have enriched for individuals based on tau pathology in addition to amyloid pathology, which can exacerbate screen failures. We explored enrichment for decliners with a model that forecasts clinical progression, AD‐Px, while minimizing screen failures and avoiding strict tau cut‐offs.

**Method:**

In amyloid‐positive early AD participants from ADNI (adni.loni.usc.edu), we quantified screen failure rates due to tau enrichment with CSF phosphorylated tau 181 (ptau‐181) measured by the Roche Elecsys assay at a 26.9 pg/mL cut‐off. That cut‐off best separated AD and cognitive unimpaired participants. We performed power analyses to estimate sample sizes for detecting 30% treatment effects at 80% power for two‐arm trials enrolling participants at this cut‐off. We compared the screen failure rates and sample size estimates of the ptau‐181 cut‐off against enrichment with decliners predicted by AD‐Px. AD‐Px is a machine learning model that uses APOE4 status, demographics, clinical assessments, and CSF biomarkers to predict individual risks of decline over 2 years. We replicated these analyses in an independent cohort of amyloid‐positive early AD participants from NACC (naccdata.org).

**Result:**

In ADNI, 36.2% of participants failed to meet the ptau‐181 cut‐off, and 453 individuals were required to power a trial. AD‐Px screened out only 16.7% of participants, while maintaining equivalent statistical power with a sample size estimate of 453. Compared to the ptau‐181 cut‐off, AD‐Px had a 53% smaller screen failure rate (p < 0.001) (Fig. 1). In NACC, the ptau‐181 cut‐off screen failed 42.1% of participants and a power analysis estimated a sample size of 518 individuals. Selecting AD‐Px decliners in NACC, however, screen failed only 11.6% patients and required a sample size of 508 individuals. In NACC, AD‐Px had a 72% smaller screen failure rate compared to ptau‐181 (p < 0.001) (Fig. 2).

**Conclusion:**

AD‐Px effectively reduces screen failure rates caused by restrictive biomarker cut‐offs, enabling more flexible inclusion criteria while maintaining statistical power. By substantially reducing screen failure rates, AD‐Px can accelerate patient enrollment, lower screening costs, and shorten trial durations without compromising sample quality.